# Balancing animal welfare and assisted reproduction: ethics of preclinical animal research for testing new reproductive technologies

**DOI:** 10.1007/s11019-018-9827-0

**Published:** 2018-02-07

**Authors:** Verna Jans, Wybo Dondorp, Ellen Goossens, Heidi Mertes, Guido Pennings, Guido de Wert

**Affiliations:** 1 Department of Health, Ethics and Society and Research School GROW for Oncology & Developmental Biology, Maastricht University, Maastricht, The Netherlands; 2 Department of Biology of the Testis (BITE), Vrije Universiteit Brussel, Brussels, Belgium; 3 Bioethics Institute Ghent (BIG), Department of Philosophy and Moral Sciences, Ghent University, Ghent, Belgium

**Keywords:** Ethics, Assisted reproductive technologies, Responsible innovation, Animal research, The three Rs

## Abstract

In the field of medically assisted reproduction (MAR), there is a growing emphasis on the importance of introducing new assisted reproductive technologies (ARTs) only after thorough preclinical safety research, including the use of animal models. At the same time, there is international support for the three R’s (replace, reduce, refine), and the European Union even aims at the full replacement of animals for research. The apparent tension between these two trends underlines the urgency of an explicit justification of the use of animals for the development and preclinical testing of new ARTs. Considering that the use of animals remains necessary for specific forms of ART research and taking account of different views on the moral importance of helping people to have a genetically related child, we argue that, in principle, the importance of safety research as part of responsible innovation outweighs the limited infringement of animal wellbeing involved in ART research.

## Introduction

Several authors have pointed out that all too often, the introduction of new ARTs has taken place on a trial and error basis, thus exposing women and their future children to potential harms of procedures that are possibly risky and of unclear benefit (Dondorp and de Wert 2011; Harper et al. 2012; Provoost et al. 2014; Van; Steirteghem 2008). There is a growing awareness of the importance of making preclinical efficacy and safety research part of the responsible introduction of new assisted reproductive technologies (ARTs). In order to achieve this, the field is called upon to heed existing guidelines (ESHRE) stating that new reproductive technologies should, where possible, be developed and tested in preclinical research using animals and/or human embryos (Pennings et al. 2007). However, whilst ensuring that new ARTs are safe and beneficial is an ethically recommendable aim, these types of research (on animals and human embryos) are also morally sensitive, thus raising the question how innovation in medically assisted reproduction can responsibly be achieved. In this paper, we will specifically address the ethics of preclinical animal research in this context.

As we will explain in the "Background" section, the recent emphasis on the need for such studies in the field of assisted reproduction coincides with simultaneous public concerns and policy efforts in international research governance to minimize the use of animals in scientific research (NC3Rs 2013; Pijnappel 2016; Ormandy et al. 2014; von Roten 2013). The European Union has even embraced the complete phasing out of animal research as a goal for European legislation (European Commission 2015). It seems that the call for more rather than less preclinical animal studies in the field of assisted reproduction is out of tune with these policy aims. The least one can say is that this call comes at a time where the justification for the use of animals in research can no longer be taken for granted. The apparent tension between these two trends underlines the urgency of an ethical exploration of whether and under what conditions research involving the preclinical development and testing of new ARTs in animals would be acceptable. Surprisingly, our study is the first to explore this specific question, thus filling an important lacuna relevant to the fields both of assisted reproduction and ethics of animal research.

We start our discussion from the assumption, also underlying the current ethical framework for animal research, that animals may be used for scientifically valid studies under the conditions of subsidiarity and proportionality (section “The ethical framework for animal research”). The first of these principles drives the question whether animals are actually needed to achieve the research aims. We address this issue in section “Alternatives for using animals to test new ARTs”. We argue that given the specific aims of testing new ARTs, a full replacement for using animals is not realistic. The second principle (proportionality) requires an analysis of the moral weight of the aims served by the research held against the moral costs in terms of animal welfare. As will be explained in section “Proportionality of using animals to test new ARTs”, two lines of argument need to be distinguished: the moral weight of helping people to have (genetically own and healthy) children and the importance of making sure that ARTs are only offered when safe and of proven benefit. As we will argue, the strength of the latter argument only counts in the light of a positive assessment of the weight of former. The final section contains concluding remarks.

## Background

### The growing awareness of the importance of preclinical research for new ARTs

As Joyce Harper and colleagues have pointed out, many new ARTs “have not been shown to be safe, to have a clear clinical benefit and/or not been properly validated” before their introduction into clinical practice, which is a “very worrying situation that may become even more common as new technologies are developed” (Harper et al. 2012). In vitro fertilization (IVF), intracytoplasmic sperm injection (ICSI), the cryopreservation of embryos, ooplasm transfer, and most recently oocyte vitrification, in vitro oocyte maturation and ‘Augment’ treatment are examples of ARTs that have been introduced into clinical practice without much preclinical research (Harper et al. 2012; Dondorp and de Wert 2011; Motluk 2015). However, the past years have seen a growing awareness of the importance of preclinical research on new ARTs.

The general lack of preclinical research has, for example, been recognized by the Task Force on Ethics and Law of the European Society of Human Reproduction and Embryology (ESHRE). In 2007, they published a statement on the welfare of the child in medically assisted reproduction and pointed out that “the widespread adoption of new techniques frequently takes place without the necessary evaluation of their efficacy (…), safety and social and economic consequences” (Pennings et al. 2007). This exposes women and children to potential risks inherent in the technologies and treatments. These risks include the potential for inducing genetic and epigenetic alterations which may result in lower developmental competence and/or health problems in offspring thus conceived. For example, research has shown that the use of different culture media leads to differences in birthweight in the resulting babies (Dumoulin et al. 2010).

### Animal research in ART

To test the efficacy and safety of new ARTs, ESHRE recommends a research chain consisting of four steps; (a) animal studies, (b) preclinical embryo research, (c) clinical trials and (d) follow-up studies (Pennings et al. 2007). This paper will focus solely on the first step; animal studies. Where animal studies are concerned, ARTs are tested at different stages of reproduction and animal development: studying gametes, preimplantation embryos, fetuses, and offspring in several generations. For these studies, animals are used in different ways: either directly: as the research model to be studied, or indirectly: as providers of those models, when animals are used as a source of gametes or as carriers of fetuses. As animals are needed also for research only looking at gametes or in vitro embryos, those studies qualify as ‘animal research’ as well.

The first steps of developing a new ART are often performed in vitro (e.g. use of human cell lines or early animal embryos). Thereafter, animal in vivo embryos, fetuses and born offspring will be tested. The nature of the precise procedures depends on the research question. For example, in the context of developing Stem Cell Derived (SCD) gametes, animal embryos created with gametes procured with this technology may be used to investigate possible epigenetic effects. After transfer to the womb, animal fetuses grown from such embryos may subsequently be used to test developmental potential. Thereafter, any resulting offspring may be tested for abnormalities, including an evaluation of their reproductive health, and of possible effects upon offspring in further generations.

The insight animal models can give in epigenetic, developmental and transgenerational effects is a significant benefit compared to other types of preclinical research (Brison et al. 2013). Moreover, in contrast to follow-up research in humans, animal follow-up research can be performed in a relatively short period. In practice, this means that multiple generations of animals generated by a new ART are observed and subjected to several tests during their lives, such as weight measurements and blood tests.

Often, rodents such as mice or rats are used for efficacy and safety research on ARTs, because of their physiological resemblance to humans, the extensive basic knowledge on these animal models and the possibility to perform transgenerational research in a short time span. When the new ART is tested successfully in lower mammals, such as mice or rats, it may be tested on other species (including non-human primates) that allow a better translation of the research outcomes to humans. For example, although mitochondrial replacement therapy (MRT) resulted in the birth of live offspring in the mouse model, when applied on human oocytes, a significant amount of mtDNA from the nuclear donor (patient) was apparently transmitted to the reconstructed embryos. Therefore, the technique would be inappropriate for patients with mtDNA-associated diseases (Tachibana et al. 2009). To investigate how the transmission of mtDNA could be prevented, researchers have used a non-human primate model, in which they successfully used spindle transfer (ST) whereby no mtDNA was transmitted (Tachibana et al. 2009).

### The three Rs as a policy aim

Simultaneous with the realization that more preclinical research, including animal research, is needed in the ART field, there is growing international support for the Three Rs (replace, reduce and refine the use of animals in research) (NC3Rs 2013; Pijnappel 2016). Many scientific journals, such as *Human Reproduction*, explicitly request that authors follow the Three Rs (Human Reproduction 2018). According to the principles of the Three Rs, researchers using animals should show that there are no other scientific methods to conduct their research and how they aim to minimize animal numbers and suffering.

In the past few years, the growing support of the Three Rs has resulted in increased regulations on animal research conform to the Three Rs (European Commission 2010; NC3Rs 2013). The 2010 European Union’s regulations on the protection of animals used for scientific purposes took full effect in 2013, and replaced the old regulations from 1986. The goal of the new Directive is to “strengthen legislation, and improve the welfare of those animals still needed to be used, as well as to firmly anchor the principle of the Three Rs, to replace, reduce and refine the use of animals, in EU legislation” (European Commission 2010). The Three Rs are directly incorporated in the Directive, as it states that “animal studies should be either replaced by methods not involving animals, or adapted to reduce the number of animals needed, or refined to minimize pain, suffering or distress experienced by the animal, or to increase their welfare. If an alternative approach to achieve a research objective is available, the Directive makes its use mandatory” (European Commission 2015). This means that, on a European level, researchers using animals have to show that they incorporate the Three Rs in order to get approval for their research. The ethical justification on this policy will be provided in the next section.

The need for more animal research in the ART field and the growing international support for the Three Rs seem to lead in opposite directions. On the one hand, there is an acknowledged need for doing more animal research as part of the responsible introduction of new ARTs while at the same time the wider message is that we should as much as possible avoid using animals for research. As both these claims refer to ethical principles, the question is how this tension can be resolved in an ethically acceptable way. How should the ART field respond to this tension and what does it mean for safety research using animals on emerging ARTs?

## The ethical framework for animal research

The acceptability of animal research is a highly debated subject in the field of bioethics as well as in the societal and political domain (Ormandy et al. 2014; von Roten 2013). The positions in this debate are often portrayed as being essentially between the biomedical and animal protection communities, diametrically opposing one another (DeGrazia 2003; Nuffield Council on Bioethics 2005). Emma Marris, reporter of *Nature*, speaks of “a ping-pong game between hard-core activists and hard-core defenders”, in which “anyone in the middle who stands up to be heard risks getting hit” (Marris 2006, p. 810). However, in that middle ground, an ethical framework has been developed for the acceptable use of animals in research for which there is in fact considerable consensus (DeGrazia 2003; Nuffield Council on Bioethics 2005).

The ethical framework recognizes that as sentient beings, animals have a moral status that needs to be taken seriously. A being has moral status, if it has interests which can be harmed (Singer 1995). The moral status of animals means that we are obliged to consider their needs and interests when making decisions affecting them (Warren 1997). Applying this to the practice of animal research, the framework insists that animals are as much as reasonably possible treated with respect to their well-being, which also includes allowing them to live the typical life of a representative of their species. As the qualifier (‘as much as reasonably possible’) indicates, not every infringement on animal wellbeing is as such regarded as reprehensible. However, recognition of their moral status entails that any such infringement requires a justification. The obvious candidate justification for animal research is that it would further the interests of humans in the development of new knowledge and better medical technologies. The framework specifies the conditions for this justification in terms of two requirements; (1) that the benefits to be derived from animal research cannot be obtained through other, less morally problematic, means (principle of subsidiarity) and (2) that the level of infringement on animal wellbeing is proportional to the moral weight of those benefits (principle of proportionality) (European Commission 2010). In practice, this means that an animal ethics committee assessing a protocol for animal research must agree that the research meets both these requirements.

Implicit in this ethical framework is the notion that animal research always comes at a moral price. Its justification therefore requires not only that, with regard to specific research protocols, this price can be shown to be the lesser of two evils (with refraining from research as the larger one), but also that accepting the practice of animal research should come with a general and continuing commitment on the part of society to as much as possible reduce (and ideally avoid) this implicit moral price. This is reflected in the concept of the 3Rs (NC3Rs 2016). Although the concept, with its emphasis on minimizing the use of animals and improving the welfare of those still being used, was already developed over fifty years ago, it is increasingly seen today as a context for overcoming the moral disagreement between the biomedical and the animal protection communities (Pijnappel 2016).

## Alternatives for using animals to test new ARTs

The principle of subsidiarity drives the question whether animals are actually needed to achieve the research aims. As discussed in the “Background” section, animal research can give insight in the risks of a certain technology and may help to reduce these risks before applying the technology to humans. Are there alternative methods with less invasive means than animal research to investigate new ARTs?

Elsewhere in medicine, alternatives for the use of animals in preclinical research have been found in computer models, the use of alternative organisms, such as lower microorganisms (e.g. yeasts), and the use of animal/human in vitro cell and tissue cultures (Doke et al. 2015). Bearing in mind the nature of ART research described in the “Background” section, it is implausible that these models can replace all ART animal research, as they cannot provide information on the development of embryos and fetuses, nor on the health of (multigenerational) offspring. For example, in studies investigating the safety of spermatogonial stem cell transplantation (SSCT) and testicular tissue grafting (TTG), there were no alternatives for animal research when testing for epigenetic effects in multigenerational offspring (Goossens et al. 2009, 2011). The use of animal/human in vitro cell and tissue cultures or the use of e.g. organoids may be an interesting option to reduce the use of animals for research (Lancaster et al. 2014). However, since many research procedures, such as performing transgenerational research, are impossible in this model, they will only have limited value.

A possibly important method to minimize the use of animals, which has not been given much attention yet as an alternative to animal research in the literature, can be research on human embryos in vitro (i.e. preclinical human embryo research). Embryo research enables researchers to test offspring for abnormalities in an early stage of life. For instance, molecular studies on preimplantation embryos can be useful to yield information on epigenetic re-programming. Moreover, the use of human embryos can also be helpful to avoid translation problems: “Preferentially and where possible, studies should be done on human embryos, as results from animal studies cannot always be extrapolated to humans” (De Rycke et al. 2002).

Proposing human embryo research as an alternative presupposes that the use of human embryos as research material is morally less problematic than that of animals, since the subsidiarity principle should be met. This, however, seems to run against the widely held view that the opposite is the case: that human embryos have a higher moral status and deserve more respect than animals. For instance, in many jurisdictions that allow human embryo research under conditions, one of those conditions is that the envisaged results cannot be obtained through other methods, including animal research. The British Warnock report explicitly states that human embryos should only be used when research with animal models is not an option (Warnock 1985). The same view is reflected in ESHRE’s account of a research chain in which human embryo studies are described as the next step, only after preceding animal studies have led to reassuring data (Pennings et al. 2007). Interestingly, the subsidiarity principle underlying the call for replacing animal research also applies to human embryo research. This means that, when holding on to the view that human embryos have a higher moral status than animals, the proposition of using human embryo research to replace animal research collides with the condition to only perform human embryo research when animal research is not an option.

We however do not find it obvious that the use of animal models is indeed morally less problematic than human embryo research. Firstly, animal research inevitably entails an infringement of animal wellbeing whereas human embryos cannot experience pain or discomfort. Secondly, as reflected in the acceptance of occasioning left-over embryos in IVF-practice, the moral status of human preimplantation embryos is relatively low (Harman 1999; Steinbock 2011; Gezondheidsraad 1998). How the conflict between these readings of the subsidiarity principle should be resolved is a matter for further debate and analysis that should also include the possible scenario of extending human embryo research beyond the current 14-day limit (Hyun et al. 2016), if that could yield important information about the safety of a new technology. To the extent that human embryo research can indeed be considered as morally less rather than more problematic than using animals for the same purpose, present accounts of the order of the ideal chain of types of preclinical safety research need to be revised. Even so, as human embryo research will not provide data beyond the earliest stages of development, it cannot be expected to provide more than a partial replacement of the use of animals for testing new ARTs.

## Proportionality of using animals to test new ARTs

The conclusion that animal research remains necessary for the responsible introduction of new ARTs is only a first step in determining the ethical acceptability of such research. The next step concerns the question whether the aims served with ART research are important enough to justify the use of animals. Whereas in the previous section we explored whether the subsidiarity principle was met, we now turn to the question of whether such research is also proportional. To answer this question, we need to explore what is in the scales on both sides of the balance. On the moral cost side, we first need to get a clearer picture of what precisely is at stake in terms of the impact on animal wellbeing where testing animals for ART research is concerned. We will then move on to the other side of the balance in order to discuss whether the moral benefits of ART research are substantial enough to outweigh the moral cost.

### The moral cost of using animals for ART research

In the “Background” section, we described the research process using animals in different stages of their lives. How do these research procedures translate to moral cost in terms of compromised animal welfare and animal suffering? To perform animal gamete or embryo research in vitro, oocytes and sperm need to be isolated from full grown animals. In some species, such as cattle, researchers can use spare material from abattoirs to retrieve gametes. In most other species, however, such as in the mouse model, females must undergo hormone stimulation. The injections lead to short and light pain for the animals, but the hormone stimulation itself does not lead to animal distress. The mice will be euthanized and their reproductive tissues removed, after which oocytes are collected from the fallopian tubes of females and sperm from the epididymis of males. Hormonal stimulation of the females is also a first step in research involving animal embryos in vivo. A few days after mating, the carrier will be euthanized and its fallopian tubes and uterus removed in order to allow the isolation of fertilized oocytes (or embryos). Fetuses and newborn animals will be euthanized prior to being tested, for instance for epigenetic abnormalities. To test the adults, a piece of tissue will be removed which may be slightly painful, but that is something that can be avoided by sedation. Thereafter, the tissue will be tested. Ultimately, most research animals will be euthanized when they are no longer useful for the research (except for e.g. nonhuman primates).

How do we evaluate the moral cost of these procedures? If one wants to assess animal research for a specific research project, the moral cost depends on what procedures are needed for answering the precise research question and concerns elements such as the number of animals used, the use of anesthetics and which animal species are needed. Here, we want to assess the moral cost of animal research on ARTs in comparison to other practices using animals. Several accepted animal research practices are highly invasive. For instance, in studies aimed at developing therapies for cancer or burn injuries, animals are exposed to conditions that may cause significant suffering as a result of tumor growth or inflicted burns (Abdullahi et al. 2014; Workman et al. 2010). Animal research on ARTs, on the contrary, is much less invasive, since animals will be at most subjected to short and light pain and are killed painlessly instead of being subjected to significant suffering. As explained by philosopher Jeff McMahan, the general view is that “it is more important to prevent the significant suffering of animals than it is to prevent, or not to cause, their deaths” (McMahan 2002). We conclude that the harm done to animals in ART research is, in comparison to cancer or burn research, relatively low, resulting in a relatively low moral cost.

Comparing animal research on ARTs to another widely accepted practice of animal use, namely the social practice of eating meat, invites the same conclusion that animal research on ART has a relatively low moral cost. Whereas animal research should always meet strict rules in accordance with the principles of the Three Rs, these principles are not incorporated by the food industry. Consequently, research animals are generally treated with more respect and are subjected to less suffering (e.g. using anesthetics, proper housing) than food industry animals.

As animal research on ARTs comes with a relatively low level of harm, the moral cost of these procedures is low in comparison with that of other generally accepted animal research practices and with the use of animals in the meat industry. Nevertheless, since also in animal research on ARTs, animals suffer some degree of pain or are killed, their use as research models is in need of justification. This leads to the question whether the moral cost is proportional to the potential benefits.

### Justifying aims of animal research: human health as a benchmark

In line with the ethical framework discussed in section “The ethical framework for animal research”, the European Directive refers to “the avoidance, prevention, diagnosis or treatment of disease, ill-health or other abnormality or their effects in human being” as justifying aims of animal research (European Commission 2010). This reflects the widely shared notion that health is such a vital need for humans that if animal research can ever be regarded as serving a sufficiently important aim, it must be for health. If we take this as the upper end of a spectrum of considered acceptability, then most cosmetics research would be found at the lower end. Longstanding ethical concerns about the proportionality of the use of animals for consumer cosmetics has led to an official ban of this practice in the entire European Union since 2013 (European Commission 2009). Apart from the availability of possible alternative methods for safety-testing, an important reason for this is that developing products that merely cater to consumer preferences is not regarded as sufficiently weighty to justify the inevitable infringement of animal wellbeing. Taking the uncontested value of health as a benchmark, our question thus becomes where on this spectrum the benefits provided by ART research are to be positioned.

This question cannot simply be answered by pointing to the fact that the relevant research is meant to protect humans from possible health risks connected to untested technologies. While this is a necessary element of any justification of the use of animals for preclinical safety studies, it is not sufficient to make the case. As with regard to consumer cosmetics, whether the use of the relevant technology would be safe only becomes a proportionality affecting issue when it is first established that the use of the technology as such serves a morally weighty aim. Therefore, the proportionality question should be addressed on two levels. We will start our assessment on the first level, where it is questioned whether the benefits of MAR are important enough to justify the use of animals. Only when this criterion can be met, the step can be made to the second level, where it is questioned whether the aims of ART *research* are important enough to justify the use of animals.

### The moral importance of medically assisted reproduction

With regard to the first level, the aim for which MAR was originally developed, i.e. helping people with fertility problems to have children, has led to a long debate about whether infertility should be regarded as a disease (Holm 1996). Reasons for an affirmative answer (Zegers-Hochschild et al. 2017) are that on the level of biological functioning, fertility problems can be ascribed to observable or presumed abnormalities in the reproductive system. Although MAR does not take away or ‘cure’ those abnormalities, it provides fertility patients with something (a child) for which reproductively healthy couples need no medical help. Clearly, for those taking this view, developing new ARTs for people with fertility problems is a matter of developing health care, putting the importance of the needed research in the higher end of the spectrum.

Others have however argued that infertility is not so much a disease, but rather a (social) handicap (Holm 1996). In their view, childlessness becomes a problem in the context of societal expectations and personal desires, rather than as a direct consequence of a biological abnormality. This also connects with the idea that for those who regard their childlessness as a problem, there are other options for having a child, thus relativizing the importance of developing new ARTs. For example, adoption is an alternative to medical treatment, or having a child through low-tech donor insemination is an alternative to high-tech ICSI. This might lead to the conclusion that the importance of developing new ARTs is relatively low. However, the value that many people place on having a child with a genetic link to (ideally) both partners, points in the opposite direction and is an important driver for developing new ARTs capable of providing just that: a child of which both partners are the genetic parents.

Currently, ARTs are not only being developed to help the infertile to have genetically related children, but also to help other people, regardless of their fertility status. Future MAR options using SCD-gametes will to a large extent serve that aim. This might also make it possible for people who are unable to reproduce due to their sexual orientation, relationship status or age to have a genetically related child. For women who for whatever reason expect not to be able to fulfill their child-wish prior to running out of functional oocytes, the development of SCD-gametes will also make oocyte or ovarian tissue cryopreservation redundant. Since it is obvious that when dealing with this wider range of requests, MAR does not respond to a health problem and that for those involved there may be alternative routes to having a child, the question becomes how the importance of these reproductive services relates to the uncontested value of human health on the one end of the spectrum and the trivial importance of consumer cosmetics on the other.

A specific form of MAR entails helping people to have children who are not only genetically related, but also healthy. This concerns people who may or may not have a fertility problem, but who want to avoid having a child with a genetic disorder that they are at risk of transmitting. MAR treatments that will allow them to have a child without the specific genetic disease include PGD, MRT, or possibly germline gene editing (CRISPR-cas9) in the future. In terms of our spectrum, treatments to avoid the transmission of genetic diseases might be regarded as falling in the category of health. The argument behind this reasoning is that a healthy child can only be realized by ensuring that the child is born without the genetic defects of which their parents are healthy or affected carriers. However, to realize the aim of having a healthy child, parents can also choose for using donor gametes or adoption instead of medical treatment. Nevertheless, many patients choose the latter option, despite the invasiveness of ART treatments. This shows, again, that there is apparently a socially determined motive for preferring the options leading to a genetically related child over other alternatives.

As it appears most appropriate to say that the practice of MAR serves the aim of helping people to have a genetically related child, our question about the moral weight of developing new ARTs requires an assessment of the importance of this aim. Although research has shown that there are no significant differences considering psychological well-being between biologically and non-biologically related parents and children (Golombok et al. 2004, 2006; Lansford et al. 2001), and although some commentators have called the preference to have genetically related children ‘irrational’ (Holm 1996; Bayles 1984), it is clear that many people do consider the genetic link to be very important. For instance, the decision to establish a family through donor conception is often only made after a long process of failed ART treatments with the prospective parents’ own gametes and coming to terms with this situation may for many be possible only after a period of serious grieving. Moreover, what are often presented as alternative ways of having a child are not always available due to strict conditions (adoption) or scarcity (donor oocytes) or come with challenges of their own. For example, the abolishment of donor anonymity in a growing number of countries (which in itself can be regarded as reflecting an increased societal emphasis on the importance of the genetic link (Pennings 2012)) has made donor conception less attractive to many couples who are weary about future contact of their child with the donor and the possible role that the donor may want to play in their family (Brewaeys et al. 2005).

We acknowledge that given the different views about the importance of the genetic link, MAR cannot be said to serve a vital human need in the same way some other medical interventions do. However, it would neither be correct to say that it serves a trivial preference. Given that those different views connect to the plurality of understandings of what a flourishing life means, and in the light of the value attached to that plurality in our liberal society, we argue that the practice of MAR does indeed serve a morally weighty aim.

Is MAR important enough to justify the use of animals in research aimed at improving the practice (if no alternative methods are available)? We see two reasons for arguing that it is. Firstly, as long as the large-scale use of animals for food, where alternatives are readily available in the form of vegetarian or vegan lifestyles, is considered morally acceptable by society, it seems difficult to maintain that research aimed at improving MAR would not be sufficiently important. Secondly, the earlier observation that, also in comparison with the use of animals in the meat industry, preclinical ART research has a relatively low impact on animal wellbeing, further supports this conclusion.

### The proportionality of preclinically testing new ARTs

If the above reasoning is sound, the argument on the next level can be more straightforward. Here we are concerned with the moral importance of preclinically testing new ARTs for efficacy and safety. Testing the efficacy of a new ART prior to its clinical introduction is important to avoid subjecting women (and sometimes men) to potentially risky treatment without sufficient benefit. Testing its safety also concerns the health of children and future generations. Although animal research can never guarantee the safety of a new technology, testing ARTs in animal models prior to introducing them in the clinic can reduce the risk that children conceived through these technologies may be born with malformations or experience health problems during the rest of their lives. The fact that only few such health effects of new ARTs have until now emerged cannot be a reason for complacency in this regard (Dondorp and de Wert 2011). As embryologist Anne McLaren has famously said, the direct clinical introduction of untested new ARTs can be compared with ‘making the first test of a new aircraft-guidance system on a crowded Boeing 747’ (McLaren 1989). As this regards human health related concerns that as such fall on the higher end of the spectrum of justificatory aims of animal research, we conclude that a strong case can be made that, in principle, the use of animals for testing new ARTs would be proportional.

## Concluding remarks

Considering that the use of animals remains necessary for specific forms of ART research and taking account of different views of the moral importance of helping people to have a genetically related child, we argue that, in principle, the importance of safety research as part of responsible innovation outweighs the limited infringement of animal wellbeing involved in ART research. In principle, because the proportionality of concrete instances of animal research still depends on the specifics of the case. As animal research inevitably comes at a moral price, it remains important to constantly consider to what extent the ideal of the 3Rs can be better met. Although a full replacement of animal research on new ARTs is not realistic, possible alternatives including the use of human embryos should be considered in concrete cases.
